# Draft Genome Sequences of Two Unclassified Bacteria, *Hydrogenophaga* sp. Strains IBVHS1 and IBVHS2, Isolated from Environmental Samples

**DOI:** 10.1128/genomeA.00884-17

**Published:** 2017-08-24

**Authors:** Russell J. S. Orr, Stephane Rombauts, Yves Van de Peer, Kamran Shalchian-Tabrizi

**Affiliations:** a Section for Genetics and Evolutionary Biology (EVOGENE), Department of Biosciences, University of Oslo, Oslo, Norway; b Department of Plant Biotechnology and Bioinformatics, Ghent University, Ghent, Belgium; c VIB Center for Plant Systems Biology, Ghent, Belgium

## Abstract

We report here the draft genome sequences of *Hydrogenophaga* sp. strains IBVHS1 and IBVHS2, two bacteria assembled from the metagenomes of surface samples from freshwater lakes. The genomes are >95% complete and may represent new species within the *Hydrogenophaga* genus, indicating a larger diversity than currently identified.

## GENOME ANNOUNCEMENT

Advances in high-throughput sequencing, coupled with decreasing costs, have led to the number of available bacterial genomes increasing almost exponentially. Genome sequencing, however, has traditionally been limited to species that can be held and grown in culture due to the high DNA volumes needed. A predominant focus on cultivable species has led to a genome bias, where the true bacterial diversity is poorly represented. Metagenomic studies are rectifying this bias and have already revealed a large novel diversity (1). However, metagenomic studies remain limited, with many ecosystems yet to be sampled. We attempt to expand species richness in a bioproject with a goal to identify novel bacteria from various environmental samples. Here, we present the draft genomes of two unclassified *Hydrogenophaga* bacteria, isolated from the surface of freshwater lakes in Norway (Årungen, Ås) and Japan (Tsukuba, Ibaraki).

DNA was isolated using a standard phenol-chloroform protocol with ethanol precipitation and subsequent cleaning using Zymo genomic clean and concentrator. DNA was prepared and sequenced on the Illumina HiSeq2500 platform (150-bp paired-end reads; 350-bp insert size) with PacBio RS2 P6-C4 chemistry (20 kb) at the Norwegian Sequencing Centre. Metagenome drafts were assembled using SPAdes version 3.9.0 (2), single genomes were separated with MetaBAT (3), and quality was assessed with CheckM (4). Separate genomes were scaffolded using LINKS (5), and gaps were closed with Sealer (6). Genome assemblies were evaluated with PROmer (7) and REAPER (8) before being improved with Pilon (9). Genomes were annotated using the NCBI Prokaryotic Genome Annotation Pipeline (10). Taxonomical rank was established on evaluation of CheckM (4), PhyloSift (11), and megaBLAST against the NCBI nr database.

*Hydrogenophaga* sp. IBVHS1 was assembled into two scaffolds, constituting seven contigs with a sequence length of 4.43 Mb and a GC content of 64.98%. The scaffold *N*_50_ was 3.05 Mb with Illumina coverage of 207× and PacBio coverage of 52×. CheckM estimated genome completeness at 97.26% with no contamination or strain heterogeneity. The genome constitutes 4,194 genes, 46 RNAs, 40 tRNAs, 4 noncoding RNAs (ncRNAs), and 46 pseudogenes.

*Hydrogenophaga* sp. IBVHS2 was assembled into seven scaffolds, constituting nine contigs with a total sequence length of 3.17 Mb and a GC content of 68.73%. The scaffold *N*_50_ was 0.96 Mb with Illumina coverage of 197× and PacBio coverage of 35×. CheckM estimated genome completeness at 95.56% with no contamination or strain heterogeneity. The genome constitutes 2,952 genes, 43 RNAs, 39 tRNAs, 3 ncRNAs, and 23 pseudogenes.

### Accession number(s).

The draft genomes of *Hydrogenophaga* sp. strains IBVHS1 and IBVHS2 sequenced under this project have been deposited at DDBJ/EMBL/GenBank under the accession numbers NFUU00000000 and NFUT00000000, respectively. These biosamples (SAMN06840507 and SAMN06840508, respectively) are part of BioProject PRJNA384425.
